# Cryoablation Without Excision for Early-Stage Breast Cancer: ICE3 Trial 5-Year Follow-Up on Ipsilateral Breast Tumor Recurrence

**DOI:** 10.1245/s10434-024-16181-0

**Published:** 2024-09-16

**Authors:** Richard E. Fine, Richard C. Gilmore, Kenneth R. Tomkovich, Jill R. Dietz, Michael P. Berry, Lydia E. Hernandez, Karen S. Columbus, Susan A. Seedman, Carla S. Fisher, Linda K. Han, Eric R. Manahan, Randy D. Hicks, Rashmi P. Vaidya, Lisa D. Curcio, Alexander B. Sevrukov, Andrew S. Kenler, Bret Taback, Margaret Chen, Megan E. Miller, Linsey Gold, Beth V. Anglin, Hussein D. Aoun, Rache M. Simmons, Sheldon M. Feldman, Susan K. Boolbol

**Affiliations:** 1 Margaret West Comprehensive Breast Center, West Cancer Center and Research Institute, Germantown, TN USA; 2 CentraState Medical Center, Freehold, NJ USA; 3 University Hospital Cleveland Medical Center, Cleveland, OH USA; 4 Cleveland, OH USA; 5 Cincinnati Breast Surgeons Inc, Cincinnati, OH USA; 6 Presbyterian Cancer Center, Rio Rancho, NM USA; 7 Indiana University IU Health Hospital-University Hospital, Indianapolis, IN USA; 8 Dalton Surgical Group, Dalton, GA USA; 9 Regional Medical Imaging, Flint, MI USA; 10 Ironwood Cancer and Research Centers, Glendale, AZ USA; 11 BreastLink Laguna Hills, Laguna Hills, CA USA; 12 Thomas Jefferson University Hospital, Philadelphia, PA USA; 13 Bridgeport Hospital, Bridgeport, CT USA; 14 Columbia University Medical Center, New York, NY USA; 15 Comprehensive Breast Care, Troy, MI USA; 16 Complete Breast Care, Plano, TX USA; 17 Karmanos Cancer Institute, Detroit, MI USA; 18 Weill Cornell Weill Medical College, Cornell University, New York, NY USA; 19 Montefiore-Einstein Center for Cancer Care, New York, NY USA; 20 Mount Sinai Beth Israel, New York, NY USA; 21 Nuvance Health System, Poughkeepsie, NY USA

## Abstract

**Background:**

The ICE3 trial evaluated the safety and efficacy of cryoablation in women aged ≥60 years with low-risk, early-stage breast cancers, aiming to provide a non-operative treatment option and avoid potential surgical risks. This study presents 5-year follow-up trial results.

**Methods:**

The ICE3 trial is an Institutional Review Board-approved, prospective, multicentered, non-randomized trial including women ≥ 60 years of age with unifocal, ultrasound visible, invasive ductal carcinoma ≤ 1.5 cm in size, histologic grade 1–2, hormone receptor (HR)-positive, and human epidermal growth factor receptor 2 (HER2)-negative. The primary study endpoint of 5-year ipsilateral breast tumor recurrence (IBTR) was evaluated based on Kaplan–Meier estimates.

**Results:**

Overall, 194 patients meeting eligibility received successful cryoablation treatment per protocol and were included for analysis. The mean age was 74.9 years (55–94) with a mean tumor size of 7.4 mm transverse (2.8–14.0 mm) and 8.1 mm sagittal (2.5–14.9 mm). With a mean follow-up period of 54.16 months, the IBTR rate at 5 years was 4.3% and breast cancer survival was 96.7%. Of the 124 patients who received endocrine therapy only, the IBTR was 3.7%. No serious device-related adverse events were reported. Minor (88.2%) and moderate (9.6%) adverse events were mild in severity and resolved without residual effects. Quality-of-life score demonstrated statistically significant improvement (*p* < 0.001) in distress at 6 months as compared with baseline.

**Conclusions:**

Breast cryoablation presents a promising alternative to surgery in selected patients, offering the benefits of a minimally invasive procedure with minimal risks. Further studies are encouraged to confirm cryoablation as a viable alternative to surgical excision low-risk patients.

In 2024, an estimated 310,720 new cases of invasive breast cancer will be diagnosed among US women.^1^ A better understanding of tumor biology has allowed a more patient centric approach in breast cancer treatment. Currently, the use of genomic profiling is influencing patient management decisions, where more favorable tumor biology allows for de-escalation in therapy.

Elderly patients with early-stage, low-risk (low grade, hormone receptor [HR]-positive, human epidermal growth factor receptor 2 [HER2]-negative) breast cancers are being offered less aggressive adjuvant treatments. Avoiding post-lumpectomy radiation (CALGB C9343,^2^ Prime II^3^) and the ability to avoid routine use of sentinel lymph node biopsy in stage I, HR-positive breast cancer patients > 70 years of age (Choosing Wisely recommendation from the Society of Surgical Oncology) are two examples of less aggressive surgery and adjuvant therapies.^4,5^ Avoiding surgical intervention altogether in early-stage, low-risk breast cancer patients may be considered in an appropriate subset of patients.

To avoid the potential risks of surgery, while maintaining efficacy associated with breast-conservation therapy, non-operative, minimally invasive ablation techniques have been evaluated. Understanding the technological innovations of cryo-systems (making extremely low temperatures stable), tumor cryoablation at a molecular level and attention to appropriate patient selection has led to cryoablation currently being considered a safe, effective, and adaptable technique.^6^

Cryoablation utilizes liquid nitrogen to achieve temperatures as low as −170 °C with a rapid freezing rate (> 100 °C/min). This method ensures complete cellular destruction at temperatures below −19 °C.^7^ Tumor destruction mechanisms include direct damage (intracellular ice formation, osmotic dehydration) and indirect damage (ischemia, immunologic response).^8–10^ Recent findings suggest an abscopal effect, where cryoablation affects cancer cells outside the ablated area.^11^ The balance between necrosis and apoptosis influences the immunomodulation induced by cryoablation.^10,12^ Studies show that immunogenic intracellular contents release activates immune cells such as cytotoxic T lymphocytes, locally and distally, generating a robust immune response with antineoplastic and prooncogenic effects.^11,13^

Cryoablation is particularly appealing because it can be performed in an office setting using local anesthesia, with better patient tolerance, improved cosmesis, and potential cost savings.^14^ The ICE3 trial aims to evaluate the efficacy and safety of cryoablation without excision for low-risk, early-stage breast cancer.

## Methods

### Study population

The ICE3 trial is an Institutional Review Board (IRB)-approved, prospective, multicentered, non-randomized trial including women ≥ 60 years of age with unifocal, ultrasound-visible invasive ductal carcinoma ≤ 1.5 cm in size and with a low-risk cancer profile, including estrogen receptor (ER) and/or progesterone receptor (PR) positivity, HER2 negativity, and a low to intermediate histology grade (Nottingham grade I–II) as confirmed by core needle biopsy. The trial was conducted in 19 sites in the US and was approved by the Western Institutional Review Board (WIRB) for 12 sites and IRBs for 7 sites.

The patients included in the trial were clinically lymph node-negative on ultrasound. The exclusion criteria ruled out patients with an extensive intraductal component (EIC; defined as a core biopsy specimen containing 25% or more of intraductal neoplasia), multifocal and/or multicentric disease, the presence of multifocal calcifications on mammogram, prior surgical biopsy for diagnosis or treatment of the index lesion, known coagulopathy or thrombocytopenia, patients not suitable for cryoablation according to the treating physician, and patients receiving neoadjuvant therapy in any form. Selected sites gained IRB approval for enrollment of patients aged 50 years or older (total of 4 patients). The first patient was enrolled in October 2014 and the last patient in February 2019. Initially, 212 patients were enrolled.

Three patients had screen failures, and 3 patients withdrew consent. Consequently, 206 patients received cryoablation treatment and subsequent follow-up evaluation. Variation from the inclusion criteria was identified after ablation for nine additional patients who were then excluded (five for a tumor size > 1.5 cm, one for EIC, one for multifocal disease, and two for previous neoadjuvant treatment before cryoablation), and 3 patients did not receive protocol-mandated treatment. Thus, 194 patients met full eligibility for the study and received successful cryoablation per protocol.

### Outcomes

Ipsilateral breast tumor recurrence (IBTR) at 5 years was the primary outcome, as defined by biopsy. Patients were followed by clinical breast examination and breast imaging at 6 months, and then annually at 12, 24, 36, 48, and 60 months after the procedure. Biopsy was performed in cases where suspicious lesions were detected.

Secondary outcomes included disease-free survival (DFS; the time between the cryoablation procedure until local, regional, or distant breast cancer recurrence), breast cancer survival (the time between cryoablation procedure until the date of death from breast cancer or up to 60 months’ follow-up visit) and overall survival (the time between cryoablation procedure until the date of death from any cause or up to the 60-month follow-up visit). Patients who died without a specified cause were considered as death related to breast cancer. The National Comprehensive Cancer Network (NCCN) Distress Tool was used before the procedure at baseline and then 6 months after cryoablation.^15^ Patients and physicians were required to rank their satisfaction with cosmetic results on a scale of 1 (very dissatisfied) to 5 (very satisfied) at each follow-up visit (at 6, 12, 24, 36, 48, and 60 months). Safety data included adverse events reporting during the course of the study (from procedure time and up to 5 years). All adverse events were classified according to the Common Terminology Criteria for Adverse Events (CTCAE) v.4.0.^16^

### Sample Size

The primary study outcome was a local IBTR at 5 years through the width of the 95% confidence interval (CI). A sample size was calculated for this outcome. For a two-sided 95% exact Clopper Pearson CI of the IBTR rate whose true value was 5%, a sample size of 150–200 patients was required to yield a half-width of 5% at most with more than 99% power. In this context, power is the probability (conditional method) of obtaining a CI a half-width less than or equal to the hypothesized value.

### Statistical Analysis

The study endpoints were evaluated based on Kaplan–Meier estimates. Survival analysis naturally accounts for differential follow-up, including staggered patient enrollment and varying dropout and event times. Subjects missing data were censored at the point of their withdrawal. The full data set (*n* = 206) was used to evaluate the safety outcomes. Per protocol analysis set (*n* = 194) was used for the primary and secondary endpoints. Observation of recurrence at any time is carried forward as a known 5-year IBTR event, while death without recurrence is carried forward as a known 5-year freedom from an IBTR event.

The protocol specified that if the upper limit of the 95% CI at the 5-year time point is < 10%, the study will be considered successful. All analyses were performed using SAS 9.4M8 (SAS Institute Inc. Cary, NC, USA).

### Cryoablation Technique

All procedures were performed using the ProSense Cryosurgical System (IceCure Medical Ltd, Caesarea, Israel). This device uses liquid nitrogen to reach cooling temperatures (−196 °C). The cryoprobe achieves rapid freezing by creating an active freeze zone up to its distal tip. An isolated zone proximal to the freeze zone prevents unwanted freezing along the cryoprobe shaft. The device achieves rapid and stable cooling alternated with slow thawing, creating an ice ball with large lethal zones. Under ultrasound guidance, the cryoprobe (140 mm/diameter 10 G) was inserted through a stab incision into the center of the lesion along the longest axis of the lesion parallel to the chest wall. Activation of the cryoablation system caused cooling of the cryoprobe to extremely low temperatures (−170 °C). This was achieved by conductive heat removal from the tissue and consequent cell destruction by freezing.

One treatment session with a double-freezing method was used for each patient. Each freezing cycle duration was determined based on the ice ball dimension along the transverse axis (ice ball width) measured under real-time ultrasound. The freeze time stopped when the ice ball reached the predetermined ablation size. According to the protocol, treatment times were defaulted to a minimum of a 9-min freeze, an 8-min passive thaw, and a second 9-min freeze.

Because the ice ball growth varies from patient to patient, treatment times were controlled at the investigator’s discretion. Treatment times were adjusted to reach at least a 35-mm ice ball at the end of the first freeze and a 40-mm ice ball at the end of the second freeze, not to exceed 12 min for either freeze cycle. The 35–40 mm ice ball size was considered necessary to create a sufficient lethal zone around the tumor with a reasonable margin based on the upper size limit of 1.5 cm for inclusion. At the end of the treatment, the cryoprobe is automatically warmed to allow extraction.

The total procedure time was 20–40 min. The site of cryoprobe insertion was determined by the physician’s preference based on lesion location, orientation, or both. Successful penetration of the cryoprobe along the longest caliber of the lesion was achieved when the distance from the distal tip of the cryoprobe to the mid portion of the lesion, where the lowest temperature is expected, was approximately 20 mm.

A cautious approach was taken to avoid thermal injury to the skin and chest wall, especially for patients with small breasts. During the freezing procedure, the cryoprobe was lifted gently to prevent frost injury to the chest wall. Ultrasonography-guided injection of saline between the skin and the ice ball anterior surface was performed to avoid frost injury to the skin. All adverse event definitions and classifications were according to the CTCAE 4.0.^16^

Per protocol, adjuvant treatment was at the discretion of the treating physician. Patients were followed by clinical breast examination and breast imaging at 6 months, and then annually at 12, 24, 36, 48, and 60 months after the procedure.

## Results

### Characteristics and Outcomes

Consistent with the ICE3 interim results, 194 patients who received successful cryoablation per protocol were eligible for follow-up in the study.^17^ The mean age of the patients was 74.9 ± 6.9 years (range 55–94 years). The mean tumor sagittal dimension was 8.1 mm (range 2.5–14.9 mm), the mean tumor transverse dimension was 7.4 mm (range 2.8–14 mm), and the mean tumor anterior-posterior dimension (A-P) was 6.3 mm (range 1–14 mm) [Table 1].

**Table 1:** Patient characteristics of eligible patients undergoing cryoablation

Patient characteristics	
Age, years	
Mean ± SD	74.9 ± 6.9
Median (range)	74.5 (55–94)
Race	
Caucasian	160 (82.5)
African American	14 (7.2)
Hispanic	12 (6.1)
Native American	2 (1.0)
Asian	1 (0.5)
Not specified/declined to answer/unknown	5 (2.6)
Tumor characteristics	
Nottingham tumor score (combine histologic grading)	
Low: 1 (range 3–5)	96 (49)
Intermediate: 2 (range 6–7)	98 (51)
Receptor status	
ER+	194 (100.0)
PR+	180 (92.8)
HER2−^a^	194 (100.0)
Tumor size by ultrasound (day of procedure)	
Mean mm ± SD	Sagittal: 8.1 ± 2.9
	Transverse: 7.4 ± 2.7
	A-P: 6.3 ± 2.6
Median mm (range)	Sagittal: 8.0 (2.5–14.9)
	Transverse: 7.0 (2.8–14)
	A-P: 6.0 (1–14)

SD standard deviation, ER estrogen receptor, PR progesterone receptor, HER2 human epidermal growth factor receptor 2, A-P anterior-posterior, FISH fluorescence in situ hybridization

^a^HER2 was tested with immunohistochemistry and, if equivocal, a FISH assay was performed

All tumors were either grade 1 or 2, ER-positive, and HER2-negative, with 92.8% also showing PR positivity. Following the second freeze, the mean dimensions of the ice ball in patients who did not experience an in-breast recurrence (IBTR) during the follow-up period was 4.7 cm in length and 3.7 cm in width.

**Fig. 1:**
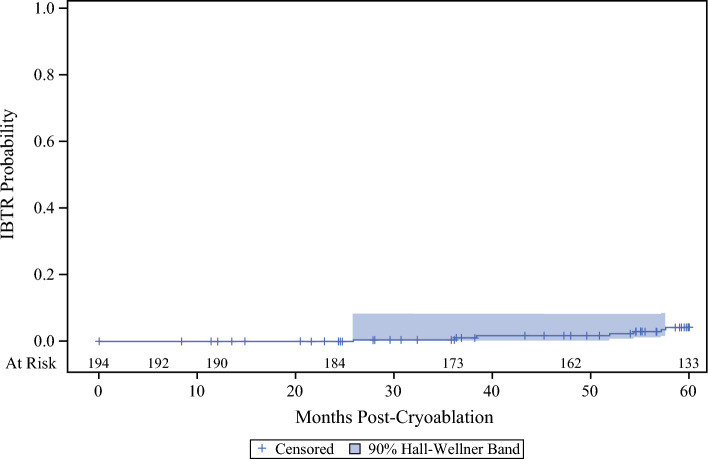
Kaplan–Meier plot of IBTR probability curve. The survival analysis IBTR rate was 4.3%, with an exact 95% CI of 2.1–8.7%. For all 194 subjects, the mean follow-up time was 54.2 ± 13.1 months, and the mean time to recurrence was 46.7 ± 12.4. IBTR ipsilateral breast tumor recurrence, CI confidence interval

At a mean follow-up period of 54.16 ± 13.07 months, the overall IBTR rate was 3.61% (7/194 patients). Based on the Kaplan–Meier estimate, the IBTR rate was 4.3% (95% CI 2.1–8.7%) at 60 months, 1.7% (95% CI 0.6–5.3%) at 48 months, and 0.6% (95% CI 0.1–3.9%) at 36 months (Fig. 1).

The characteristics of the 7 patients who met the inclusion eligibility criteria, received complete cryoablation treatment, and subsequently experienced recurrence are shown in Table 2. The mean time to recurrence was 46.72 months (range 25.88–63.15 months) and the mean baseline tumor size at the largest dimension was 0.87 cm (range 0.58–1.38 cm). After the second freeze, the ice ball length was 4.93 cm (range 4.0–5.8 cm) and the ice ball width was 3.75 cm (range 2.98–4.39 cm). Three of the seven recurrences received no adjuvant treatment and four received endocrine therapy alone.

**Table 2:** Characteristics of eligible patients undergoing cryoablation with local recurrence

Patient characteristics	Patient 1	Patient 2	Patient 3	Patient 4	Patient 5	Patient 6	Patient 7
Age	73	67	72	72	86	79	70
Time to recurrence (months)	54.38	51.90	25.88	36.20	38.38	57.16	63.15
Nottingham grade	2	2	2	2	2	2	2
Estrogen receptor	Positive	Positive	Positive	Positive	Positive	Positive	Positive
Progesterone receptor	Positive	Positive	Positive	Negative	Positive	Positive	Positive
Max tumor size on procedure day (cm)	0.99	0.60	0.58	0.58	1.30	1.38	0.64
Ice ball length (cm)	5.0	4.0	5.0	4.0	5.8	5.7	5.0
Ice ball width (cm)	4.1	3.1	3.3	3.0	4.2	4.	4.4
SLNB	No	No	No	No	No	No	No
Adjuvant radiation (Y/N)	No	No	No	No	No	No	No
Adjuvant chemotherapy (Y/N)	No	No	No	No	No	No	No
Adjuvant endocrine therapy (Y/N)	Yes	No	No	Yes	Yes	No	Yes

SLNB sentinel lymph node biopsy, Y yes, N no

In addition to the seven reported ipsilateral recurrence cases, 2 patients had distant metastasis (one also had ipsilateral recurrence) and 4 patients had second primary breast cancer, with no regional recurrence, with a 5-year DFS rate of 92.8% (95% CI 87.6–95.8%), based on the survival analysis. The breast cancer survival rate was 96.7% (95% CI 92.2–98.6%), where 2 patients died due to distant metastasis from breast cancer and three for unknown reasons. The overall survival rate was 88.6% (82.9–92.5%), where all remaining patients died from comorbidities not related to breast cancer.

Of the 194 patients included in the study, 153 received adjuvant treatment, among whom 2.61% had a recurrence (4/153). Specifically, 124 patients (63.9%) received endocrine treatment only, 3 (1.5%) underwent whole-breast radiation only, 25 (12.9%) received endocrine plus radiation, and 1 patient (0.5%) received endocrine, radiation, and chemotherapy.

**Fig. 2:**
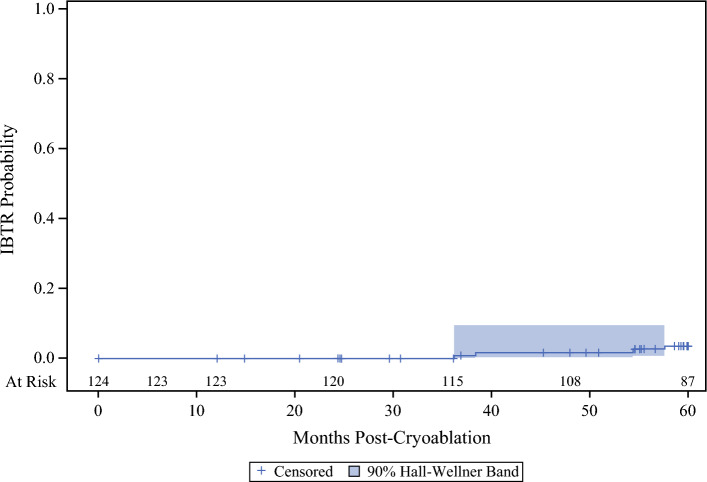
Subgroup Kaplan–Meier plot of IBTR probability curve. For the endocrine therapy only subgroup (*n* = 124), the survival analysis IBTR rate was 3.7%, with an exact 95% CI of 1.4–9.6%. The mean time to recurrence was 48.0 ± 11.2. IBTR ipsilateral breast tumor recurrence, CI confidence interval

Among the endocrine therapy-only subgroup, 4/124 (3.2%) had an IBTR, with a survival analysis IBTR rate of 3.7% (95% CI 1.4–9.6%) [Fig. 2], DFS of 94.9% (95% CI 88.1–97.9%), breast cancer survival of 96.1% (95% CI 89.9–98.5%), and overall survival of 89.5% (95% CI 82.2–93.9%).

Sentinel node biopsy was performed for 19 patients after cryoablation. One patient with a positive sentinel node received radiation and endocrine therapy. This patient had not experienced a recurrence at the 60-month follow-up evaluation of the study.

### Quality of Life

All cryoablation procedures performed were in the outpatient setting and all patients were discharged on the same day. The patients reported an average of 1.7 ± 1.5 days to resume normal activity. Of the cohort with data available (181/194), 82.9% (150/181) returned to their full daily activities 48 h after the procedure.

Patients and physicians reported 100% cosmetic satisfaction at 5 years follow up, ranging from 99.3% to 100% for the patients (*n* = 177 and *n* = 111, respectively, of the 194 patients) and from 98.6% to 100% for the physicians (*n* = 176 and *n *= 102 physician satisfaction reports, respectively, of the 194 patients/procedures) at the 6 months to 5 years follow-up visits.

Statistically significant improvement in patients’ distress was demonstrated using the NCCN Distress Tool at 6 months as compared with baseline (*p* < 0.001) from a median score of 4.0 to 2.0.

### Early Withdrawal

As anticipated in an aging population, there was an expected rate of withdrawal due to advanced age and related comorbidities. Follow-up was also complicated by the impact of coronavirus disease 2019 (COVID-19), especially among patients within the study’s target age group. Indeed, of 194 patients, 16 patients died of reasons unrelated to breast cancer, including heart failure, respiratory failure, myocardial infarction, cardiac arrest, non-traumatic intracerebral hemorrhage, and renal failure leading to multiorgan failure. Additionally, 32 patients were lost to follow-up or withdrew and were censored in the Kaplan–Meier estimates from their last clinical visit.^17^

### Safety Evaluation

No serious adverse events related to the study or procedure were reported. Each investigator was requested to report adverse events and to determine their relationship to the device or procedure (no or yes, and if yes, possible, probable, or remote relationship to the device (Table 3). All serious adverse events reported during the study were found to have no relation to the study device or procedure. Rather, they were related to the advanced age of the subjects and their comorbidities, including urinary tract infection (UTI), stroke, respiratory failure, and pneumonia. Overall, 187 device-related non-serious adverse events were reported by 97 patients (Table 3). All adverse events were resolved, and the patients fully recovered without experiencing residual effects. Most adverse events (88.2%) were mild in severity (CTCAE grade 1) and included bruising (25.7%), pain in needle insertion (20.9%), localized edema (19.3%), local hematoma (4.3%), tenderness (4.3%), pruritis and rash (2.1%), erythema (1.6%), minor skin freeze burn (1.6%), injection site reaction (1.6), drainage (1.1%), fatigue (1.1%), hemorrhage (0.5%), skin induration (0.5%), skin infection (0.5%), local skin flushing (0.5%), tethering (0.5%), dimpling (0.5%), breast twitches (0.5%), and heat sensation (0.5%). Moderate adverse events (9.6%) [CTCAE grade 2] included bruising (5.3%), edema (1.6%), freeze-related skin burns (1.1%), which resolved with topical treatment, pain (1.1%) and local hematoma (0.5%), and severe bruising (2.1%, CTCAE grade 3) were reported, all resolved without residual effects (Table 4).

**Table 3:** Device-related adverse events (*n* = 206)

No. of AEs	187

Seriousness classification			
Serious		Non-serious	
0	0%	187	100%

Intensity classification					
Mild		Moderate		Severe	
165	88.2%	18	9.6%	4	2.1%

Relation to study					
Remotely		Possible		Probable	
7	3.7%	28	14.9%	152	81.3%

AEs adverse events

**Table 4:** Non-serious device-related adverse events

	*N*	%
*Mild*		
Bruising	48	25.7
Pain	39	20.9
Edema	36	19.3
Hematoma	8	4.3
Tenderness	8	4.3
Pruritus and rash	4	2.1
Erythema multiforme	3	1.6
Injection site reaction	3	1.6
Burn	3	1.6
Fatigue	2	1.1
Drainage	2	1.1
Flushing	1	0.5
Skin infection	1	0.5
Breast twitches	1	0.5
Heat sensation	1	0.5
Breast warm to the touch	1	0.5
Tethering	1	0.5
Dimpling	1	0.5
Hemorrhage	1	0.5
Induration at cryo site	1	0.5
*Moderate*		
Bruising	10	5.3
Edema	3	1.6
Burn	2	1.1
Pain	2	1.1
Hematoma	1	0.5
*Severe*		
Bruising	4	2.1
*Total non-serious*		
	187	100

## Discussion

### Cryoablative Success

Cryoablation has been used for treating tumors in various organs, including the lung,^18,19^ kidney,^20^ and liver,^21,22^ among others.^23^ Clinical experience demonstrated that cryoablation is precise, safe, and effective.^21^ The treatment of breast diseases with cryoablation started with benign breast lesions (fibroadenoma) in 1987 and increased rapidly after 2000.^24–29^ Successful results of cryoablation treatment for malignant breast cancer have been demonstrated in animal models.^30,31^ Imaging findings in the hours and days after cryoablation, show uniform necrosis throughout the previously frozen area. Over time, the granulation tissue shrinks,^6,24–27^ leaving scar tissue and fat necrosis that reabsorbs over time.^17^

Studies by Sabel et al.,^32^ Manenti et al.,^33,34^ and the American College of Surgeons Oncology Group (ACOSOG)^35^ highlighted the efficacy of cryoablation for small breast tumors. Sabel et al.^32^ reported 100% success in cryoablation for invasive ductal carcinomas sized 1 cm or smaller, and complete ablation for tumors up to 1.5 cm, excluding those with invasive lobular or significant ductal carcinoma in situ (DCIS).

Manenti et al.^33,34^ corroborated these findings in post-menopausal women with breast cancers smaller than 2 cm, achieving successful lesion destruction (in two series, 14/15 patients in one, and 38/40 in the other), with confirmation of destruction by follow-up breast magnetic resonance imaging (MRI) and pathologic assessment of the surgically excised treated area.

The ACOSOG phase II study reported a 75.9% success rate (by pathology) for tumors under 2 cm, rising to 93.8% for tumors sized 1 cm or smaller, and 92% when excluding multifocal disease. All of the tumors sized 1 cm or smaller were successfully cryoablated when multifocal disease was excluded.^35^

Collectively, these studies underscore the high success rate of cryoablation for small, localized breast tumors, particularly those 1 cm or smaller.

### Local Recurrence

In the study conducted by Adachi et al.^36^ in a similar patient population (*n* = 193), only one (1/193, 0.5%) patient experienced a recurrence after the treatment of invasive carcinoma 12 months post cryoablation.

In 2021, Habrawi et al.^37^ described the results of percutaneous cryoablation (PCA) used for women with infiltrating ductal carcinomas, ER/PR-positive, and HER2-negative sized 1.5 cm or smaller. Most of the patients were older than 60 years of age. One cryoprobe was used per patient. All tumors had a 1- to 2-cm freeze margin past the tumor to ensure the complete ablation of tumor tissue. None of the patients had serious complications. They all tolerated the procedure well with minimal discomfort, and no-one required pain medication other than over-the-counter pain relievers. The most common post-procedure complaint was breast pain (soreness), bruising, and edema. No cosmetic deficits were reported. Of 12 patients, 11 had a 6-month follow-up evaluation at the time of publication, and 4/12 patients a had 2-year follow-up evaluation. None had evidence of disease recurrence. As in the current study, the authors suggested that early breast cancers up to 15 mm in size with a favorable low-risk profile can be safely and effectively treated with a single session of cryoablation performed in the office without the need for subsequent surgery.

In 2023, Khan et al.^38^ enrolled 32 patients with a diagnosis of clinically node-negative, ER/PR-positive, HER2-negative infiltrating ductal carcinomas 1.5 cm or smaller and a mean age of 71 years. For all patients, adjuvant endocrine therapy was recommended and 6 patients (18.75%) received adjuvant radiation. Of the 32 patients, 20 (60.6%) have been followed-up for 2 years or longer, with no residual or recurrent disease at the site of ablation. They reported that cryoablation of the primary tumor without undergoing sentinel node biopsy provides an oncological safe and feasible minimally invasive office-based treatment option instead of surgery for patients with early-stage, low-risk breast cancer.

Kawamoto et al.^39^ reported the results of ultrasound-guided PCA for early-stage breast cancer. In their study, 18 patients with breast tumors ranging in size from 6 to 14.5 mm were treated with cryoablation. There was no local recurrence or metastasis during the 5-year follow-up.

In the CALGB 9343 trial, Hughes et al.^2^ randomized women age 70 years or older with HR-positive clinical T1N0 breast cancers undergoing lumpectomy to either tamoxifen with whole-breast radiation therapy (RT) or tamoxifen alone. There were significant differences between the two groups. Specifically, in the group that omitted RT, there was a higher proportion of women aged 80 years and older, single women, women with tumors 0–1 cm, and women with two or more comorbidities (*p* < 0.001). In addition, there were notable disparities between the groups in terms of their geographic regions. At the 5-year follow-up evaluation, RT improved the locoregional recurrence rates (1% for RT vs. 4% for no RT) but did not confer a survival advantage. The 10-year survival data demonstrated no significant difference in time to distant metastasis, whereas breast cancer-specific survival and overall survival were similar in the two groups.^2^

Kunkler et al. studied a similar cohort of patients age 65 years or older with HR-positive tumors sized 3 cm or smaller undergoing lumpectomy who were randomized to receive either endocrine therapy with whole-breast RT or endocrine therapy alone.^3,37^ The cumulative incidence of local breast cancer recurrence within 10 years was 9.5% (95% CI 6.8–12.3) in the no radiotherapy group and 0.9% (95% CI 0.1–1.7) in the radiotherapy group. Although local recurrence was more common in the group that did not receive radiotherapy, the 10-year incidence of distant recurrence as the first event was not higher in the no radiotherapy group than in the radiotherapy group, at 1.6% (95% CI 0.4–2.8) and 3.0% (95% CI 1.4–4.5), respectively. The 10-year overall survival rates were almost identical in both groups, with 80.8% (95% CI 77.2–84.3) for those without radiotherapy and 80.7% (95% CI 76.9–84.3) for those with radiotherapy. No significant difference was observed in the rates of regional recurrence and breast cancer-specific survival between the two groups. While forgoing radiotherapy was linked to a higher occurrence of local recurrence, it did not negatively impact distant recurrence as the initial event, or overall survival in women aged 65 years and above with low-risk, HR-positive early breast cancer.

These prospective randomized trials demonstrated local-regional recurrence rates, for patients receiving endocrine therapy without whole-breast radiation, of 4% at 5 years. Our 3-year interim analysis of the primary outcome suggested an IBTR was on track to be similar to that in the aforementioned breast-conservation trials.^17^ Moreover, the longer 5-year follow-up evaluation demonstrates these local-regional recurrence rates remained consistent between these patient cohorts. Notably, subgroup analysis showed only four recurrences among patients prescribed endocrine therapy after cryoablation (124 patients) for an ipsilateral recurrence, with a survival analysis IBTR rate of 3.2%.

Post-cryoablation imaging findings, caused by the unique mechanism of tissue distraction, can persist for varying periods. Recognizing these findings and differentiating them from residual or recurrent cancer is important. These findings, such as scarring and fat necrosis, typically decrease over time, with significant changes during follow-up imaging at 6-, 12-, and 24-month intervals on mammography and ultrasound. Huang et al.^6^ and Thai et al.^40^ noted that while initial imaging may show pronounced changes, these often become less conspicuous, allowing for reliable surveillance over time. Pigg and Ward^41^ highlighted that the presence of scar tissue and granulation can mimic or obscure recurrent disease; however, adherence to strict imaging protocols and follow-up biopsies in suspicious cases, as performed in our study, ensures accurate detection of any IBTR. Kawamoto et al.^39^ also demonstrated that careful interpretation of MRI and mammography post-cryoablation can mitigate the impact of these artifacts, ensuring effective long-term surveillance. Thus, while cryoablation introduces certain imaging challenges, these can be managed with appropriate imaging techniques and protocols.

### Safety and Cosmesis

This study demonstrates cryoablation to be safe for early-stage breast cancer ≤ 1.5 cm, with minor adverse events (such as bruising, minor bleeding, and pain with injection of local anesthetic) similar to those associated with core needle biopsy of the breast. In addition, 100% of both the patients and the treating physicians were satisfied with the cosmetic outcome.

Multiple studies^39–42^ reported cryoablation under ultrasound guidance was found to be safe, with minor adverse events and has a high satisfaction rate and excellent cosmetic outcomes. Van de Voort et al.^42^ analyzed thermal ablation as an alternative to surgical resection. The conducted meta-analysis (*n* = 1266 patients) showed an 85% complete ablation rate in the cryoablation-specific cohort with the lowest complication rates (5%) compared with the other types of ablation(up to 18%). As with our results, cosmetic outcomes were satisfactory to excellent for most patients (> 95%).

Khan et al.^14^ compared cost effectiveness and patient-reported quality-of-life factors in 34 women with early-stage, low-risk infiltrating ductal carcinomas ≤1.5 cm who underwent cryoablation versus resection. The BREAST-Q survey, completed at least 1 year after the procedure, showed significantly better well-being with cryoablation in the physical, sexual, and breast satisfaction (cosmetic perception) domains compared with lumpectomy.

In their review, Pigg and Ward^41^ shared the current evidence that shows that cryoablation offers a safe and effective treatment option for selected patients, providing comparable oncological outcomes to traditional treatments while minimizing invasiveness and preserving breast aesthetics. Patient selection criteria, procedural techniques, and imaging follow-up protocols have been developed to optimize the effectiveness and safety of cryoablation.

In a recent review by Thai et al.,^40^ the advantages of cryoablation over surgery in breast cancer treatment were thoroughly examined. The review emphasized the crucial factors of patient selection, potential complications, and the importance of precise technique in cryoablation procedures. Analyzing nine publications, including the ICE3 study, the review identified that the ideal candidates for cryoablation were those with unifocal invasive ductal carcinoma tumors that are low grade, HR-positive, and ≤ 1.5 cm in size. The primary takeaway from their analysis was that PCA therapy represents a safe and effective alternative to breast-conserving surgery for select patients with small, low-risk tumors and promising prognoses.

### Study Limitations

This study was constrained by its industry-sponsored, single-arm, and non-randomized nature, which may have introduced selection bias and potential confounders. The study limitations are detailed in the ICE3 study interim result, with specifics regarding small tumor size in the eligible patients and the variation among physicians regarding the size of the ice ball for treatment.^17^

Additionally, adjuvant therapies received, including radiation, chemotherapy, and endocrine therapy, were at the discretion of the treating physician and were therefore not standardized. Nevertheless, a subgroup analysis was added to the current manuscript, allowing for a more unified population and demonstrating lower IBTR for cryoablation plus endocrine therapy.

It is also important to recognize that the distress tool may capture receipt of treatment in general and not the cryoablation procedure specifically, and is therefore a limitation of this tool.

## Conclusions

Our 5-year analysis of the trial’s primary outcome, IBTR at 5 years, suggests that cryoablation is safe and effective for patients with low-risk, early-stage breast cancer. During the 5-year follow-up period, IBTR in our low-risk breast cancer cohort showed local control similar to that with surgical standard of care while avoiding the potential risks of a surgical procedure. Cryoablation may be considered as an alternative to lumpectomy in this select population if followed by appropriate adjuvant treatment. Furthermore, future study within a clinical trial or registry is encouraged to confirm cryoablation as a viable alternative to surgical excision.

## References

[1] American Cancer Society. Facts and figures for 2024. Available at: https://acsjournals.onlinelibrary.wiley.com/doi/epdf/10.3322/caac.21820. Accessed 6 May 2024.

[2] Hughes KS, Schnaper LA, Bellon JR, et al. Lumpectomy plus tamoxifen with or without irradiation in women age 70 years or older with early breast cancer: long-term follow-up of CALGB 9343. *J Clin Oncol*. 2013;31:2382–7. 10.1200/JCO.2012.45.2615. 23690420 10.1200/JCO.2012.45.2615 PMC3691356

[3] Kunkler IH, Williams LJ, Jack WJ, Cameron DA, Dixon JM. PRIME II investigators. Breast-conserving surgery with or without irradiation in women aged 65 years or older with early breast cancer (PRIME II): a randomised controlled trial. *Lancet Oncol*. 2015;16:266–73. 10.1016/S1470-2045(14)71221-5. 25637340 10.1016/S1470-2045(14)71221-5

[4] Hughes KS, Schnaper LA, Berry D, et al. Lumpectomy plus tamoxifen with or without irradiation in women 70 years of age or older with early breast cancer. *N Engl J Med*. 2004;351(10):971–7. 10.1056/NEJMoa040587. 15342805 10.1056/NEJMoa040587

[5] The Society of Surgical Oncology Encourages Doctors. Five things physicians and patients should question. Last update on 13 November 2020. Available at: https://www.surgonc.org/wp-content/uploads/2020/11/SSO-5things-List_2020-Updates-11-2020.pdf.

[6] Huang ML, Tomkovich K, Lane DL, Katta R, Candelaria RP, Santiago L. Breast cancer cryoablation fundamentals past and present: technique optimization and imaging pearls. *Acad Radiol*. 2023;30(10):2383–95. 10.1016/j.acra.2023.05.019. 37455177 10.1016/j.acra.2023.05.019 PMC11826490

[7] Pusceddu C, Paliogiannis P, Nigri G, Fancellu A. Cryoablation in the management of breast cancer: evidence to date. *Breast Cancer (Dove Med Press)*. 2019;11:283–92. 10.2147/BCTT.S197406. 31632134 10.2147/BCTT.S197406 PMC6791835

[8] Baust JG, Bischof JC, Jiang-Hughes S, et al. Re-purposing cryoablation: a combinatorial ‘“therapy”’ for the destruction of tissue. *Prostate Cancer Prostatic Dis*. 2015;18(2):87–95. 10.1038/pcan.2014.54. 25622539 10.1038/pcan.2014.54

[9] Baust JG, Gage AA, Bjerklund Johansen TE, Baust JM. Mechanisms of cryoablation: clinical consequences on malignant tumors. *Cryobiology*. 2014;68(1):1–11. 10.1016/j.cryobiol.2013.11.001. 24239684 10.1016/j.cryobiol.2013.11.001 PMC3976170

[10] Sabel MS, Nehs MA, Su G, Lowler KP, Ferrara JLM, Chang AE. Immunologic response to cryoablation of breast cancer. *Breast Cancer Res Treat*. 2005;90(1):97–104. 10.1007/s10549-004-3289-1. 15770533 10.1007/s10549-004-3289-1

[11] Chen J, Qian W, Mu F, Niu L, Du D, Xu K. The future of cryoablation: an abscopal effect. *Cryobiology*. 2020;97:1–4. 10.1016/j.cryobiol.2020.02.010. 32097610 10.1016/j.cryobiol.2020.02.010

[12] Sabel MS, Su G, Griffith KA, Chang AE. Rate of freeze alters the immunologic response after cryoablation of breast cancer. *Ann Surg Oncol*. 2010;17(4):1187–93. 10.1245/s10434-009-0846-1. 20033323 10.1245/s10434-009-0846-1

[13] Aarts BM, Klompenhouwer EG, Rice SL, Imani F, Baetens T, Bex A, Horenblas S, Kok M, Haanen JBAG, Beets-Tan RGH, Gómez FM. Cryoablation and immunotherapy: an overview of evidence on its synergy. *Insights Imaging*. 2019;10(1):53. 10.1186/s13244-019-0727-5. 31111237 10.1186/s13244-019-0727-5 PMC6527672

[14] Khan SY, Snitman A, Habrawi Z, Crawford S, Melkus MW, Layeequr Rahman R. The role of cryoablation in breast cancer beyond the oncologic control: COST and breast-Q patient-reported outcomes. *Ann Surg Oncol*. 2023;30(2):1029–37. 10.1245/s10434-022-12570-5. 36171531 10.1245/s10434-022-12570-5

[15] Permissions Distress Tool. NCCN. Available at: https://www.nccn.org/guidelines/submissionslicensing-and-permissions/permissions-distress-tool. Accessed 13 May 2021.

[16] US Department of Health and Human Services. Common Criteria for Adverse Events (CTCAE)Version 4.0. 2009. Available at: https://evs.nci.nih.gov/ftp1/CTCAE/CTCAE_4.03/Archive/CTCAE_4.0_2009-05-29_QuickReference_8.5x11.pdf.

[17] Fine RE, Gilmore RC, Dietz JR, et al. Cryoablation without excision for low-risk early-stage breast cancer: 3-year interim analysis of ipsilateral breast tumor recurrence in the ICE3 trial. *Ann Surg Oncol*. 2021;28(10):5525–34. 10.1245/s10434-021-10501-4. 34392462 10.1245/s10434-021-10501-4

[18] Inoue M, Nakatsuka S, Yashiro H, et al. Percutaneous cryoablation of lung tumors: feasibility and safety. *J Vasc Intervent Radiol*. 2012;23(3):295–302. 10.1016/j.jvir.2011.11.019. 10.1016/j.jvir.2011.11.019 22265246

[19] Xu Z, Wang X, Ke H, Lyu G. Cryoablation is superior to radiofrequency ablation for the treatment of non-small cell lung cancer: a meta-analysis. *Cryobiology*. 2023;112:104560. 10.1016/j.cryobiol.2023.104560. 37499964 10.1016/j.cryobiol.2023.104560

[20] Miki K, Shimomura T, Yamada H, et al. Percutaneous cryoablation of renal cell carcinoma guided by horizontal open magnetic resonance imaging. *Int J Urol*. 2006;13(7):880–4. 10.1111/j.1442-2042.2006.01432.x. 16882047 10.1111/j.1442-2042.2006.01432.x

[21] Ei S, Hibi T, Tanabe M, et al. Cryoablation provides superior local control of primary hepatocellular carcinomas of < 2 cm compared with radiofrequency ablation and microwave coagulation therapy: an underestimated tool in the toolbox. *Ann Surg Oncol*. 2015;22(4):1294–300. 10.1245/s10434-014-4114-7. 25287439 10.1245/s10434-014-4114-7

[22] Khanmohammadi S, Behnoush AH, Akhlaghpoor S. Survival outcomes and quality of life after percutaneous cryoablation for liver metastasis: a systematic review and meta-analysis. *PLoS ONE*. 2023;18(8):e0289975. 10.1371/journal.pone.0289975. 37585405 10.1371/journal.pone.0289975 PMC10431656

[23] Igarashi K, Yamamoto N, Shirai T, et al. The long-term outcome following the use of frozen autograft treated with liquid nitrogen in the management of bone and soft-tissue sarcomas. *Bone Jt J*. 2014;96-B(4):555–61. 10.1302/0301-620X.96B4.32629. 10.1302/0301-620X.96B4.32629 24692627

[24] Whitworth PW, Rewcastle JC. Cryoablation and cryolocalization in the management of breast disease. *J Surg Oncol*. 2005;90(1):1–9. 10.1002/jso.20201. 15786430 10.1002/jso.20201

[25] Kaufman CS, Bachman B, Littrup PJ, et al. Cryoablation treatment of benign breast lesions with 12-month follow-up. *Am J Surg*. 2004;188(4):340–8. 10.1016/j.amjsurg.2004.06.025. 15474424 10.1016/j.amjsurg.2004.06.025

[26] Kaufman CS, Rewcastle JC. Cryosurgery for breast cancer. *Technol Cancer Res Treat*. 2004;3(2):165–75. 10.1177/153303460400300209. 15059022 10.1177/153303460400300209

[27] Golatta M, Harcos A, Pavlista D, et al. Ultrasound-guided cryoablation of breast fibroadenoma: a pilot trial. *Arch Gynecol Obstet*. 2015;291:1355–60. 25408274 10.1007/s00404-014-3553-5

[28] Sheth M, Lodhi U, Chen B, Park Y, McElligott S. Initial institutional experience with cryoablation therapy for breast fibroadenomas: technique, molecular science, and post-therapy imaging follow-up. *J Ultrasound Med*. 2019;38(10):2769–76. 10.1002/jum.14980. 30843236 10.1002/jum.14980

[29] Plaza MJ, Kumar AV, Sanchez-Gonzalez MA. Safety and efficacy of ultrasound-guided cryoablation for benign breast fibroepithelial lesions. *J Breast Imaging*. 2019;1(4):324–8. 10.1093/jbi/wbz047. 38424801 10.1093/jbi/wbz047

[30] Carriero S, Lanza C, Pellegrino G, et al. Ablative therapies for breast cancer: state of art. *Technol Cancer Res Treat*. 2023;22:15330338231157192. 10.1177/15330338231157193. 36916200 10.1177/15330338231157193 PMC10017926

[31] Staren ED, Sabel MS, Gianakakis LM, et al. Cryosurgery of breast cancer. *Arch Surg*. 1997;132(1):28–33. 10.1001/archsurg.1997.01430250030005. 9006549 10.1001/archsurg.1997.01430250030005

[32] Sabel MS, Kaufman CS, Whitworth P, et al. Cryoablation of early-stage breast cancer: work-in-progress report of a multi-institutional trial. *Ann Surg Oncol*. 2004;11(5):542–9. 10.1245/ASO.2004.08.003. 15123465 10.1245/ASO.2004.08.003

[33] Manenti G, Scarano AL, Pistolese CA, et al. Subclinical breast cancer: minimally invasive approaches: our experience with percutaneous radiofrequency ablation vs cryotherapy. *Breast Care*. 2013;8(5):356–60. 10.1159/000355707. 24415989 10.1159/000355707 PMC3861851

[34] Manenti G, Perretta T, Gaspari E, et al. Percutaneous local ablation of unifocal subclinical breast cancer: clinical experience and preliminary results of cryotherapy. *Eur Radiol*. 2011;21(11):2344–53. 10.1007/s00330-011-2179-2. 21681574 10.1007/s00330-011-2179-2

[35] Simmons RM, Ballman K, Cox C, et al. A phase II trial exploring the success of cryoablation therapy in the treatment of invasive breast carcinoma: results from ACOSOG (Alliance) Z1072. *Ann Surg Oncol*. 2016;23(8):2438–45. 10.1245/s10434-016-5275-3. 27221361 10.1245/s10434-016-5275-3 PMC5433250

[36] Adachi T, Machida Y, Fukuma E, Tateishi U. Fluorodeoxyglucose positron emission tomography/computed tomography findings after percutaneous cryoablation of early breast cancer. *Cancer Imaging*. 2020;20(1):49. 10.1186/s40644-020-00325-y. 32678029 10.1186/s40644-020-00325-y PMC7364607

[37] Habrawi Z, Melkus MW, Khan S, et al. Cryoablation: a promising nonoperative therapy for low-risk breast cancer. *Am J Surg*. 2021;221(1):127–33. 10.1016/j.amjsurg.2020.07.028. 32788081 10.1016/j.amjsurg.2020.07.028

[38] Khan SY, Cole J, Habrawi Z, Melkus MW, Layeequr Rahman R. Cryoablation allows the ultimate de-escalation of surgical therapy for select breast cancer patients. *Ann Surg Oncol*. 2023;30(13):8398–403. 10.1245/s10434-023-14332-3. 37770723 10.1245/s10434-023-14332-3 PMC10625946

[39] Kawamoto H, Tsugawa K, Furuya Y, et al. Percutaneous ultrasound-guided cryoablation for early-stage primary breast cancer: a follow-up study in Japan. *Breast Cancer*. 2024;31(4):695–704. 10.1007/s12282-024-01584-4. 38678120 10.1007/s12282-024-01584-4 PMC11194206

[40] Thai JN, Sevrukov AB, Ward RC, Monticciolo DL. Cryoablation therapy for early-stage breast cancer: evidence and rationale. *J Breast Imaging*. 2023;5(6):646–57. 10.1093/jbi/wbad064. 38141236 10.1093/jbi/wbad064

[41] Pigg N, Ward RC. Cryoablation for the treatment of breast cancer: a review of the current landscape and future possibilities. *Acad Radiol*. 2023;30(12):3086–100. 10.1016/j.acra.2023.06.030. 37596141 10.1016/j.acra.2023.06.030

[42] Van de Voort E, Struik GM, Birnie E, Moelker A, Verhoef C, Klem T. Thermal ablation as an alternative for surgical resection of small (≤ 2 cm) breast cancers: a meta-analysis. *Clin Breast Cancer*. 2021. 10.1016/j.clbc.2021.03.004. 33840627 10.1016/j.clbc.2021.03.004

